# An international tournament of risk factors for radical intentions: evidence from 40 countries

**DOI:** 10.3389/fpsyg.2026.1855329

**Published:** 2026-09-10

**Authors:** Jocelyn J. Bélanger, Angel Gómez, Daniel Snook, Jais Adam-Troian, Michael Wolfowicz, Shadi Akariya, Jannis Kreienkamp, Justin König, Maximilian Agostini, Alexander P. Landry, N. Pontus Leander

**Affiliations:** 1 Humanities and Social Sciences, Carnegie Mellon University in Qatar, Doha, Qatar; 2 Department of Social and Organizational Psychology, Universidad Nacional de Educacion a Distancia, Madrid, Spain; 3 Department of Psychology, Florida Gulf Coast University, Fort Myers, FL, United States; 4 School of Social Sciences, Heriot-Watt University Dubai, Dubai, United Arab Emirates; 5 Institute of Criminology, Hebrew University of Jerusalem, Jerusalem, Israel; 6 The Dataflow Company, Franconia, Germany; 7 Stanford Graduate School of Business, Stanford University, Stanford, CA, United States; 8 Department of Psychology, Wayne State University, Detroit, MI, United States

**Keywords:** moral neutralization, obsessive passion, perceived discrimination, radical intentions, violent extremism

## Abstract

**Introduction:**

Understanding the risk factors linked to violent extremism remains a central challenge in radicalization research. This study examined which risk factors are most robustly associated with radical intentions–the willingness to use violence in support of an ideology.

**Methods:**

Using general-population samples from 40 countries (*N* = 10,407) and a complementary sample of incarcerated jihadists (*N* = 80), we placed the 12 strongest established correlates identified in Wolfowicz’s (2021) meta-analysis into direct multivariate competition. Analyses included conditional random forests, multilevel models, and random-effects meta-analyses of country-specific estimates.

**Results:**

Across analytic methods and ideological targets, three risk factors consistently retained robust independent associations with radical intentions: moral neutralization (cognitive justification of violence), obsessive passion (rigid, ego-involving commitment), and perceived discrimination (subjective group-based injustice). These associations were stable within and across countries and largely invariant across ideological domains. In the incarcerated jihadist sample, moral neutralization and obsessive passion remained consistent correlates of radical intentions, whereas perceived discrimination showed a weaker and less certain association.

**Discussion:**

Collectively, these findings identify a moral–motivational–grievance combination that is robustly associated with radical intentions across ideologies. We situate this combination within major theoretical frameworks–including the 3N model, Moghaddam’s staircase model, Borum’s four-stage model, and social-movement frameworks–to clarify how these robust correlates align with constructs emphasized in theories of radicalization. The findings suggest testable prevention hypotheses concerning moral justifications for violence, rigid ideological engagement, and experiences of discrimination.

## Introduction

The persistence of violent radicalization worldwide has intensified scholarly and policy interest in identifying factors associated with willingness to engage in political violence (Institute for Economics and Peace, 2025). Although only a minority of people who endorse extremist beliefs engage in violence, radical intentions—the willingness to use violence for an ideological cause—are widely regarded as a proximal antecedent of violent behavior (McCauley and Moskalenko, 2017). This focus is consistent with foundational insights from behavioral psychology, particularly the theory of planned behavior, which identifies behavioral intentions as the most immediate predictors of action (Ajzen, 1991; Ajzen and Fishbein, 1980). Accordingly, identifying the risk factors associated with radical intentions has become central to radicalization research and prevention.

A major step toward clarifying this landscape was the meta-analysis conducted by Wolfowicz et al. (2021). From a broad array of 37 risk and protective factors, the authors identified 12 correlates of radical intentions with moderate to large pooled-correlations (*r* = 0.33–0.52), whereas the remaining 25 factors showed only very small to small correlations. The present study deliberately focuses on these 12 strong correlates because they showed the most robust and sizable empirical associations with radical intentions in the most comprehensive synthesis of the literature to date. These risk factors span multiple theoretical traditions and levels of analysis: cognitive mechanisms (e.g., moral neutralization), motivational dynamics (e.g., obsessive passion and commitment), grievance-based perceptions (e.g., perceived discrimination and relative deprivation), identity-centered processes (e.g., identity fusion and in-group superiority), emotional factors (e.g., anger and negative affect), and behavioral indicators (e.g., past activism).^1^ Rather than supporting a single explanatory framework, this meta-analysis underscores the multiplicity of factors implicated in the willingness to engage in violence in the name of a cause.

By distilling the literature to a shortlist of 12 robust correlates, the meta-analysis enables a shift toward theory building on their relative importance and interrelationships, beyond accumulating isolated risk factors. However, key limitations remain: (1) correlates were assessed independently, obscuring joint contributions amid overlaps; (2) the evidence is heavily Western-centric and often ideology-nonspecific, constraining generalizability; and (3) samples exclude individuals with verified histories of ideological violence, leaving unclear whether these correlates extend to those who have engaged in such violence. Together, these gaps highlight the need for analytic approaches that adjudicate among competing risk factors across ideological domains, national contexts, and levels of proximity to ideological violence.

The present study addresses the gaps by placing the strongest established correlates of radical intentions into direct competition using a combination of multivariate modeling and cross-context comparative analyses. Its primary basis is a set of large general-population samples from 40 countries; as a smaller and necessarily preliminary extension, it additionally examines a complementary sample of incarcerated extremists to ask, tentatively, whether these correlates extend to individuals who have engaged in ideological violence. The study thus aims to clarify which risk factors retain the most robust independent associations with radical intentions.

## Literature review: Wolfowicz’s meta-analysis

Wolfowicz et al. (2021) represents a rare point of empirical convergence in a literature long characterized by a proliferation of constructs and inconsistent findings. By synthesizing evidence from 127 studies comprising 206 samples, the meta-analysis extracted over 1,000 effect sizes spanning more than 100 risk and protective factors associated with radical attitudes, radical intentions, and radical behaviors. For radical intentions—the willingness to use violence in support of an ideological cause—the synthesis identified 37 statistically significant correlates.

Although Wolfowicz et al. (2021) is the most comprehensive quantitative synthesis to focus specifically on radical intentions, it is not the only relevant synthesis in this area. Other work has examined overlapping but distinct questions—for example, predictors of political-violence outcomes among adolescents and young adults (Jahnke et al., 2022), protective factors against extremism and violent radicalization (Lösel et al., 2018), and empirically derived pathway models spanning religiously motivated, right-wing, and left-wing radicalization (Pfundmair et al., 2026). We take Wolfowicz et al. (2021) as our primary point of reference because it provides pooled effect-size estimates for the broadest set of candidate risk factors specifically for radical intentions, which is precisely what a direct, competitive comparison of those factors requires; these other works either target different outcomes, focus on protective factors, or examine radicalization pathways rather than providing pooled estimates for the broad candidate set of risk factors assessed here.

The Wolfowicz et al. (2021) synthesis is especially useful for the present study because the distribution of effect sizes was highly uneven. Of the 37 correlates, twelve demonstrated moderate to large pooled-associations (*r* = 0.33–0.52), clearly separating themselves from the remainder of the field. These strongest correlates were identity fusion (*r* = 0.52), obsessive passion (*r* = 0.50), radical attitudes (*r* = 0.48), negative affect (*r* = 0.47), activism intent (*r* = 0.44), commitment (*r* = 0.43), anger (*r* = 0.40), in-group superiority (*r* = 0.37), perceived discrimination (*r* = 0.37), moral neutralization (*r* = 0.36), collective relative deprivation (*r* = 0.36), and past activism (*r* = 0.33). This reduction—from over one hundred examined variables to a small set of robust risk factors—substantially narrowed the explanatory space and placed meaningful constraints on viable theories of radical intentions.

At the same time, the composition of this shortlist presents a clear theoretical challenge. The 12 strongest correlates do not reflect a single explanatory tradition but instead represent a diverse array of psychological correlates drawn from competing theoretical frameworks in radicalization research. Cognitive variables such as moral neutralization—cognitive processes that enable individuals to justify or rationalize violent behavior, thereby disengaging from their personal moral standards (Bandura, 1999)—align with theories of moral disengagement. Motivational constructs such as obsessive passion—a rigid, compulsive form of engagement in which the cause controls the person—and commitment—the extent to which an individual is psychologically dedicated to a cause—reflect the Dualistic Model of Passion (Bélanger, 2021; Rip et al., 2012; Vallerand et al., 2003). Grievance-based variables, including perceived discrimination and collective relative deprivation, draw on social-grievance frameworks such as relative deprivation theory, which emphasize subjective injustice as a catalyst for political violence (Guimond et al., 2025; Gurr, 1970). Identity-based processes such as identity fusion and in-group superiority capture the alignment of personal and collective identity, increasing readiness to sacrifice for the group and to justify intergroup aggression (Gómez et al., 2020; Swann et al., 2012). Finally, affective correlates such as anger and negative affect reflect emotional drivers emphasized in accounts of radicalization and political violence (Borum, 2011).

This heterogeneity is theoretically informative, but it also introduces an interpretive problem. If variables indexing grievances, emotions, identity, motivation, and moral cognition all show substantial bivariate associations with radical intentions, then radicalization may appear to be multiply determined by a wide array of risk factors. Yet the meta-analytic evidence also suggests that these correlates are not independent. Several are conceptually adjacent and plausibly causally linked, raising the possibility that the strongest factors reflect interacting components of a smaller number of underlying processes rather than 12 distinct pathways.

One implication of this interdependence is that the theoretical meaning of any single risk factor depends on what it is allowed to compete with. For instance, grievance-based perceptions may intensify negative affect and anger; identity fusion may amplify commitment and obsessive passion; and moral neutralization may reduce moral restraint once radical attitudes are adopted. From this perspective, some correlates may function as upstream antecedents, others as downstream expressions, and others still as enabling or gatekeeping processes. We note, however, that the present design does not test these mediational or sequential pathways among the predictors directly; rather, by placing the constructs into direct competition, it estimates which retain independent, non-redundant associations with radical intentions once shared variance is taken into account, leaving the causal architecture among them for future longitudinal and experimental work. Without such competition, it remains unclear which correlates represent foundational factors and which primarily reflect correlated byproducts of broader processes.

The meta-analysis also raises questions of scope and generalizability. Although Wolfowicz et al. (2021) synthesized evidence from multiple countries, the empirical base is concentrated in a relatively narrow set of contexts. The study focused on 20 OECD countries, with approximately 56% of samples drawn from Europe (e.g., Denmark, Spain, the United Kingdom) and a further 27% from the United States. This emphasis reflects a deliberate focus on democratic contexts but introduces potential Western bias by excluding regions such as the Middle East and North Africa, which experience disproportionately high levels of extremist violence (Institute for Economics and Peace, 2025). As a result, it remains unclear whether the relative importance and organization of the strongest correlates generalize to settings characterized by different political structures, conflict dynamics, and cultural norms.

Relatedly, the meta-analysis examined heterogeneity across ideological domains, including left-wing, right-wing, religious, and nonspecific extremism. Statistically significant differences were identified for only six factors—for example, stronger associations between moral neutralization and left-wing extremism, prayer frequency and right-wing extremism, and legitimacy perceptions and religious extremism. However, theoretical interpretation of these differences is constrained by the composition of the underlying evidence base. Approximately 40% of the primary studies did not focus on any specific ideological movement, limiting insight into how distinct motivational, cultural, and normative logics shape radicalization processes across ideologies. Consequently, the meta-analysis clarifies which correlates are robust on average but leaves unresolved whether their theoretical roles and relative importance vary systematically across ideological contexts.

Finally, the evidence synthesized by Wolfowicz et al. (2021) is drawn exclusively from general population, student, or at-risk samples. Individuals with verified histories of political violence are notably absent from studies of radical intentions. This creates further theoretical ambiguity. Many models implicitly assume continuity between the formation of radical intentions and the enactment of political violence (e.g., Kruglanski et al., 2019; Moghaddam, 2005). If the strongest correlates of radical intentions do not generalize to individuals who have engaged in violence, then theories may need to distinguish more clearly between factors driving endorsement of violence from those driving the transition to action.

Taken together, the meta-analysis provides both empirical clarity and a sharpened theoretical agenda. It identifies a limited set of strong correlates of radical intentions, but leaves unresolved questions about their interrelationships, relative importance, generalizability, and relevance to individuals closest to ideological violence. The next step is therefore not to expand the list of risk factors, but to adjudicate among established candidates and clarify which are most central to radical intentions.

## The present study

The present study addresses the core limitations identified in prior synthesis by moving from broad accumulation to systematic adjudication of the most robust risk factors of radical intentions. Building on Wolfowicz et al. (2021) meta-analysis, we focus on the twelve correlates that showed moderate to large pooled-correlations (*r* = 0.33–0.52): identity fusion, obsessive passion, radical attitudes, negative affect, activism intent, commitment, anger, in-group superiority, perceived discrimination, moral neutralization, collective relative deprivation, and past activism.

The study places these established risk factors into direct multivariate competition to determine which retain the most robust independent associations when the risk factors’ overlapping effects are taken into account, across widely varying contexts, and in samples that differ in proximity to extremist violence. To this end, we employ three complementary analytic strategies: (1) conditional random forests, which rank variable importance while accommodating correlations and nonlinearities; (2) multilevel regression models, which decompose within- and between-sample associations while accounting for clustering; and (3) random-effects meta-analyses of country-specific estimates, which quantify cross-context consistency and heterogeneity.

The primary dataset consists of general-population samples drawn from 40 countries, strategically selected to extend beyond the Western/OECD-centric evidence base of prior syntheses (Wolfowicz et al., 2021) by spanning OECD and non-OECD contexts, including representation from the Middle East and North Africa, and covering multiple ideological domains (left-wing, right-wing, and religious). To assess whether risk factors identified in general-population samples extend to individuals with verified histories of ideological violence, the study also includes a complementary sample of incarcerated jihadist offenders in Spain. This high-risk sample offers a rare, if preliminary, opportunity to examine whether correlates of radical intentions identified in general-population samples also characterize individuals who have engaged in ideological violence.

By triangulating evidence across analytic approaches, national contexts, ideological domains, and proximity to violence, the study aims to: (1) identify a parsimonious set of risk factors that consistently correlate with radical intentions when placed in direct competition; (2) assess the stability of these factors across diverse contexts; and (3) evaluate the extent to which they generalize to individuals who have engaged in ideological violence.

In doing so, the study aims to resolve major theoretical ambiguities from prior synthesis—especially the relative importance, interdependence, and cross-contextual generalizability of the strongest risk factors—and to offer a clearer empirical basis for understanding the psychological correlates of radical intentions. Although we examined heterogeneity across countries, ideologies, and proximity to violence, we treat these comparisons—particularly the cross-ideological one—as secondary and exploratory, with no strong a priori expectations for substantial differences in the relative importance of these processes.

## Materials and methods

### Procedure

We conducted a cross-national online survey in 40 countries between January and May 2023 using Qualtrics Panel Management. We aimed to recruit approximately 250 respondents per country, a sample size shown to yield stable correlation estimates for medium-sized effects in psychological research (Schönbrodt and Perugini, 2013). To ensure that ideology-tailored items referred to participants’ own in-group, recruitment in each context was restricted to self-identified supporters or adherents of the focal group (screened *via* panel-provider screening questions). In Muslim-majority countries, the focal group was Islam; in India, Hinduism; in all other countries, the focal group was the primary governing political party during the data collection window. In the United States, we fielded two separate partisan samples (Democrats and Republicans), yielding 41 samples across 40 countries. All questionnaires were translated into the dominant language of each participating country using a translation/back-translation procedure conducted by professional translators.

After providing informed consent (Institutional Review Board protocol number masked for review), participants completed demographic questions (age, gender, education, and religion) and an attention check early in the survey. Participants then completed the scales described below, after which they were debriefed and thanked for their participation.

In Spain, we additionally collected the 42nd sample, consisting of 80 incarcerated jihadist offenders (*N* = 80). Participants completed an adapted version of the questionnaire administered within correctional facilities. For comparability with the cross-national survey, analyses were restricted to measures that overlapped with the cross-national instrument, with radical intentions serving as the dependent variable (consistent with the general-population samples). Specifically, radical intentions were measured with two preregistered items from the same scale used in the general-population samples (e.g., “Using any means, even violent ones, to help the cause of Islam.”), worded to reference the cause of Islam—consistent with the item wording used in the Muslim-majority-country general-population samples rather than a governing political party. This approach aligns with prior studies that have assessed cognitive outcomes, including radical intentions, in offender samples (e.g., Gómez et al., 2021; Webber et al., 2018). Notably, while the sample consists of individuals with verified histories of ideological violence, the focus remains on current radical intentions rather than past offending, allowing evaluation of whether risk factors generalize across differing levels of proximity to ideological violence.

### Preregistration

The general population study and the incarcerated sample study were preregistered prior to data collection. The preregistration for the general population study is available at: https://osf.io/ex98u/overview?view_only=39d8b9fd10004297a04a2cfbd8aa81bc. The preregistration for the incarcerated sample study is available at: https://osf.io/ywtgm/overview?view_only=692110d95b524ac597f54a7b05a7cf6d. Both preregistrations include research materials and planned analyses. Analysis code is available at: https://extremism.blindreview.org/.

### Participants

In total, 10,407 respo ndents were included in the cross-national analyses (*M*_*age*_ = 36.97, *SD*_*age*_ = 13.43); 45.4% identified as women and 54.6% as men. Education was assessed on an 8-point scale (1 = *Primary*, 2 = *Secondary*, 3 = *Vocational*, 4 = *Bachelor*, 5 = *Higher*, 6 = *Master*, 7 = *PhD*, 8 = Not answered; *M* = 4.00, *SD* = 1.40). Sample-specific demographic descriptives are reported in Table 1.

**TABLE 1:** Demographic descriptives by sample.

Sample/Group	Sample size	Gender	Age	Education	Religion
Algeria/Muslim	252	72.6/27.4	*M* = 33.32 (SD = 10.79)	*M* = 4.29 (SD = 1.60)	0.0/100.0/0.0/0.0/0.0/0.0/0.0
Argentina/Frente de Todos	260	58.1/41.9	*M* = 37.37 (SD = 10.42)	*M* = 3.52 (SD = 1.27)	64.6/0.4/30.0/0.4/1.9/0.0/2.7
Australia/Australian Labor Party	257	32.3/67.7	*M* = 41.90 (SD = 17.14)	*M* = 3.99 (SD = 1.52)	35.8/7.4/48.2/0.4/1.2/0.0/7.0
Austria/Österreichische Volkspartei	242	62.8/37.2	*M* = 35.80 (SD = 11.84)	*M* = 4.46 (SD = 1.36)	44.6/8.3/37.6/2.5/5.0/0.0/2.1
Brazil/Partido dos Trabalhadores	255	52.9/47.1	*M* = 37.45 (SD = 10.55)	*M* = 4.02 (SD = 1.52)	88.2/0.0/7.8/0.0/0.0/0.0/3.9
Canada/Liberal Party of Canada	241	36.1/63.9	*M* = 47.92 (SD = 17.60)	*M* = 3.75 (SD = 1.39)	51.0/4.6/33.2/4.1/1.7/0.0/5.4
Colombia/Partido Liberal Colombiano	256	60.2/39.8	*M* = 34.22 (SD = 11.31)	*M* = 3.72 (SD = 1.33)	68.0/0.8/14.5/0.8/0.4/0.0/15.6
Czech/ANO 2011	258	55.0/45.0	*M* = 37.64 (SD = 15.36)	*M* = 3.40 (SD = 1.41)	32.2/0.4/63.6/0.4/1.6/0.0/1.9
Denmark/Socialdemokratiet	233	46.4/53.6	*M* = 38.55 (SD = 13.98)	*M* = 3.59 (SD = 1.64)	59.2/4.3/34.8/0.9/0.0/0.0/0.9
Egypt/Muslim	260	68.1/31.9	*M* = 32.82 (SD = 9.61)	*M* = 4.05 (SD = 1.02)	0.0/100.0/0.0/0.0/0.0/0.0/0.0
Finland/Sosialidemokraattinen puolue	255	47.5/52.5	*M* = 37.36 (SD = 15.42)	*M* = 3.67 (SD = 1.52)	62.7/2.0/33.7/0.0/0.0/0.0/1.6
France/La République En Marche	169	49.7/50.3	*M* = 45.20 (SD = 17.36)	*M* = 4.07 (SD = 1.52)	51.5/17.2/26.0/0.6/1.2/0.0/3.6
Germany/Sozialdemokratische Partei Deutschland	255	67.8/32.2	*M* = 36.62 (SD = 12.10)	*M* = 4.09 (SD = 1.07)	43.5/14.1/38.8/0.0/2.7/0.0/0.8
Greece/Nea Dimokratía	258	46.5/53.5	*M* = 37.72 (SD = 11.72)	*M* = 4.31 (SD = 1.41)	92.2/1.2/6.2/0.4/0.0/0.0/0.0
Hungary/Fidesz-KDNP	258	39.5/60.5	*M* = 38.71 (SD = 12.91)	*M* = 3.37 (SD = 1.32)	76.7/1.2/17.8/0.0/1.6/0.0/2.7
India/Hindu	255	60.8/39.2	*M* = 29.53 (SD = 8.54)	*M* = 4.68 (SD = 1.25)	24.7/51.4/9.0/1.2/4.3/0.0/9.4
Israel/Likud supporters	259	66.0/34.0	*M* = 36.20 (SD = 12.96)	*M* = 4.01 (SD = 1.49)	9.3/1.9/1.9/86.1/0.4/0.0/0.4
Italy/Fratelli d’Italia	255	49.0/51.0	*M* = 40.59 (SD = 13.38)	*M* = 4.62 (SD = 1.27)	83.1/1.6/12.5/0.4/1.6/0.0/0.8
Jordan/Muslim	257	70.8/29.2	*M* = 33.28 (SD = 10.01)	*M* = 3.92 (SD = 1.21)	0.0/100.0/0.0/0.0/0.0/0.0/0.0
Kuwait/Muslim	249	69.5/30.5	*M* = 32.63 (SD = 8.72)	*M* = 4.31 (SD = 1.30)	0.0/100.0/0.0/0.0/0.0/0.0/0.0
Lebanon/Muslim	243	63.0/37.0	*M* = 31.14 (SD = 9.99)	*M* = 3.88 (SD = 1.50)	0.0/100.0/0.0/0.0/0.0/0.0/0.0
Malaysia/Muslim	258	48.1/51.9	*M* = 33.84 (SD = 8.89)	*M* = 3.76 (SD = 1.23)	0.0/100.0/0.0/0.0/0.0/0.0/0.0
Mexico/Movimiento Regeneración Nacional	255	59.6/40.4	*M* = 34.22 (SD = 10.18)	*M* = 4.04 (SD = 1.05)	58.4/0.0/23.9/0.8/0.8/0.0/16.1
Morocco/Muslim	259	76.4/23.6	*M* = 33.02 (SD = 11.00)	*M* = 4.13 (SD = 1.45)	0.0/100.0/0.0/0.0/0.0/0.0/0.0
Netherlands/Volkspartij voor Vrijheid en Democratie	249	66.7/33.3	*M* = 36.55 (SD = 11.96)	*M* = 4.25 (SD = 1.04)	65.1/4.8/25.7/0.4/1.6/0.0/2.4
Nigeria/Muslim	252	67.1/32.9	*M* = 30.65 (SD = 8.01)	*M* = 4.27 (SD = 1.00)	0.0/100.0/0.0/0.0/0.0/0.0/0.0
Norway/Arbeiderpartiet	245	48.6/51.4	*M* = 33.47 (SD = 11.81)	*M* = 3.64 (SD = 1.58)	42.4/7.8/47.3/0.4/0.4/0.0/1.6
Pakistan/Muslim	247	66.4/33.6	*M* = 29.42 (SD = 9.34)	*M* = 4.68 (SD = 1.23)	0.0/100.0/0.0/0.0/0.0/0.0/0.0
Philippines/Lakas–Christian Muslim Democrats	258	54.7/45.3	*M* = 32.35 (SD = 10.79)	*M* = 3.97 (SD = 1.06)	88.0/5.4/1.6/0.4/0.0/0.0/4.7
Poland/Prawo i Sprawiedliwość	259	35.1/64.9	*M* = 35.62 (SD = 12.92)	*M* = 4.04 (SD = 1.72)	90.3/0.0/5.8/0.8/1.5/0.0/1.5
Portugal/Partido Socialista	258	56.2/43.8	*M* = 40.19 (SD = 12.94)	*M* = 3.62 (SD = 1.45)	75.2/0.8/22.5/0.0/0.0/0.0/1.6
Romania/Partidul Social Democrat	257	51.8/48.2	*M* = 37.49 (SD = 14.86)	*M* = 4.04 (SD = 1.36)	94.9/0.4/1.6/0.0/0.0/0.0/3.1
Saudi Arabia/Muslim	260	58.1/41.9	*M* = 33.12 (SD = 8.78)	*M* = 3.94 (SD = 1.22)	0.0/100.0/0.0/0.0/0.0/0.0/0.0
Spain/Partido Socialista Obrero Español (PSOE)	257	45.1/54.9	*M* = 39.00 (SD = 12.66)	*M* = 3.89 (SD = 1.38)	66.1/2.3/30.0/0.0/0.0/0.0/1.6
Sweden/Sveriges Socialdemokratiska arbetareparti	255	47.1/52.9	*M* = 39.39 (SD = 14.43)	*M* = 3.44 (SD = 1.42)	48.6/9.0/39.2/0.0/0.8/0.0/2.4
Thailand/Pheu Thai Party	259	49.4/50.6	*M* = 36.09 (SD = 9.58)	*M* = 3.87 (SD = 0.89)	0.4/2.7/2.3/0.4/93.4/0.0/0.8
Tunisia/Muslim	247	71.3/28.7	*M* = 33.26 (SD = 8.60)	*M* = 4.62 (SD = 1.20)	0.0/100.0/0.0/0.0/0.0/0.0/0.0
Turkey/Adalet ve Kalkınma Partisi	260	43.1/56.9	*M* = 34.19 (SD = 9.63)	*M* = 3.82 (SD = 1.14)	0.8/97.3/1.5/0.0/0.0/0.0/0.4
United Kingdom/Conservative Party	256	43.4/56.6	*M* = 51.22 (SD = 16.81)	*M* = 3.70 (SD = 1.40)	62.9/2.7/29.7/0.8/0.8/0.0/3.1
US Democrats/Democrats	250	38.0/62.0	*M* = 48.41 (SD = 15.84)	*M* = 4.50 (SD = 1.69)	67.6/5.6/16.8/4.8/0.8/0.0/4.4
US Republicans/Republicans	259	21.6/78.4	*M* = 51.15 (SD = 17.26)	*M* = 4.21 (SD = 1.63)	69.9/0.8/24.7/0.8/1.2/0.0/2.7
Spain/Incarcerated jihadist offenders	80	100.0/0.0	*M* = 36.45 (SD = 10.69)	*M* = 1.81 (SD = 1.07)	0.0/100.0/0.0/0.0/0.0/0.0/0.0

Cells in the Gender and Religion columns list percentages only. For Gender the order is: Male/Female. For Religion the order is: Christian/Muslim/No religion/Jewish/Buddhist/Hindu/Other (Other aggregates any remaining categories). Values are percentages and may not sum exactly to 100 due to rounding. Education shows the mean and standard deviation of a numeric scale (*Primary* = 1, *Secondary* = 2, *Vocational* = 3, *Bachelor* = 4, *Higher* = 5, *Master* = 6, PhD = 7, *Not Answered* = 8). For the incarcerated jihadist sample, age was not recorded for 2 participants and education for 6; education was assessed with a four-level item mapped onto Primary, Secondary, and Higher, so its mean is not directly comparable to the cross-national samples.

### Scales

Table 2 summarizes all measures (construct, scale source, number of items, example item, response format, and internal consistency). Unless otherwise noted, items were rated on 7-point agreement scales (1 = *Strongly disagree*, 7 = *Strongly agree*), and scale scores were computed as the mean of constituent items.

**TABLE 2:** Measures and descriptive statistics.

Construct	Scale/source	Items (n)	Example item	Response scale	*M* (*SD*)	α
Moral neutralization	Ribeaud and Eisner (2010)	16	“It is alright to fight to protect your friends.”	1–7	2.17 (1.22)	0.95
Anger	Kassinove et al. (1997)	10	“I feel infuriated when I do a good job and receive a poor evaluation.”	1–6	2.84 (1.01)	0.90
Positive affect	Watson et al. (1988)	5	“Enthusiastic”	1–5	3.61 (0.75)	0.80
Negative affect	Watson et al. (1988)	5	“Afraid”	1–5	2.25 (0.89)	0.84
Commitment	Bélanger et al. (2022)	4	“The Liberal Party of Canada is important for me.”	1–7	3.87 (1.79)	0.89
Collective relative deprivation	Obaidi et al. (2019)	6	“Supporters of the Liberal Party of Canada should have the same opportunities to improve their lives as others have.”	1–7	3.28 (1.50)	0.87
Identity fusion	Doosje et al. (2012) 2013	7	“I am one with the cause of the Liberal Party of Canada.”	1–7	3.59 (1.82)	0.95
In-group superiority	Doosje et al. (2012) 2013	4	“I believe that supporters of the Liberal Party of Canada are better people than people who endorse another political party.”	1–7	3.58 (1.85)	0.91
Obsessive passion	Bélanger et al. (2022)	6	“I have almost an obsessive feeling for being involved in the Liberal Party of Canada.”	1–7	2.87 (1.72)	0.92
Harmonious passion	Bélanger et al. (2022)	6	“Being involved in the Liberal Party of Canada is in harmony with the other things in my life.”	1–7	3.79 (1.85)	0.95
Activist intent	Moskalenko and McCauley (2009)	4	“I would join or belong to an organization that fights for the Liberal Party of Canada’s rights.”	1–7	3.07 (1.80)	0.93
Past activism	Moskalenko and McCauley (2009), Wong et al. (2019)	4	“Invited a friend to attend a meeting of a Liberal Party of Canada organization or event.”	Yes/No	0.39 (0.38)	0.81
Perceived discrimination	Geiger and Brewster (2018)	11	“People have talked down to me because I support the Liberal Party of Canada.”	1–6	2.03 (1.29)	0.97
Radical attitudes	Bélanger et al. (2019a)	3	“It is acceptable to use violence to further a cause.”	1–7	1.91 (1.69)	0.94
Intuitive–logical reasoning	Research Team	2	“I rely on my intuition when making decisions.”	1–7	3.72 (1.85)	NA
Peaceful intent	Adapted from Gousse-Lessard et al. (2013)	6	“Participate in a peaceful demonstration for the Liberal Party of Canada.”	1–7	3.43 (2.00)	0.95
Violent intent (outcome)	Adapted from Gousse-Lessard et al. (2013)	6	“Physically attack people who ridicule or oppose the Liberal Party of Canada.”	1–7	2.13 (1.73)	0.95

Example items use the locally relevant in-group label (here: the Liberal Party of Canada). Unless otherwise noted, items used agreement response scales where higher values indicate stronger endorsement. Descriptives are pooled across samples; NA = not available (e.g., too few items).

The survey began with moral neutralization and anger, followed by the Positive and Negative Affect Schedule (PANAS), because these measures contain no ideology-specific content and therefore assess general moral reasoning and affect prior to the ideology-tailored measures. The remaining ideology-tailored measures were then presented in randomized order to minimize order effects.

All ideology-tailored measures inserted the label of the focal ideological group for the relevant context (e.g., Islam, Hinduism, or the governing political party). Because recruitment was restricted to supporters of the focal group, first-person formulations (e.g., “The [focal group] is important for me”) were meaningful across contexts.

### Analytic strategy

To identify risk factors of radical intentions that are both strong and generalizable, we triangulated evidence from three complementary approaches applied to the cross-national dataset. First, conditional random forests rank risk factors by how much they improve out-of-sample performance while allowing non-linear relationships and correlated factors. Second, multilevel regression models provide interpretable effect estimates while accounting for clustering within samples and separating within-sample from between-sample associations. Third, random-effects meta-analyses of sample-specific regression slopes quantify cross-context consistency and heterogeneity. Where applicable, we examined raw, grand-mean centered, and within-sample centered specifications; conclusions converged across specifications. Each sample was additionally classified a priori by ideological domain and entered as two orthogonal contrasts (right-wing vs. left-wing; religious vs. left-wing) in all conditional random forest and multilevel models, allowing us to test whether ideological domain itself was associated with radical intentions once the focal risk factors were accounted for (see text footnote 2).

For the incarcerated sample, we used two complementary analyses that parallel this logic. We first conducted an exploratory penalized regression (LASSO) in which risk factors competed under regularization, identifying a sparse set of comparatively robust signals. We then benchmarked the focal risk factors’ slopes against the pooled cross-national estimates to evaluate generalization to an incarcerated extremist sample.

## Results

### Measurement model checks

Before testing the risk factors, we tested whether the multi-item measures operated similarly across contexts. We conducted multi-group confirmatory factor analyses to test configural and metric invariance across the 41 general-population samples. Because our primary goal was to compare associations (not mean levels) across contexts, metric invariance (equal factor loadings) was the key criterion.

Across measures, the intended factor structures were recovered, and constraining factor loadings resulted in only small changes in incremental fit, supporting metric invariance for the primary scales. Detailed model fits, sensitivity checks, and any exceptions are provided in Supplementary File.

### Risk factors of radical intentions in the cross-national samples

To identify correlates of radical intentions that are strong, interpretable, and generalizable, we triangulated evidence from three complementary approaches: (1) Conditional random forests provide a tournament-style ranking of risk factors based on their contribution to out-of-sample model performance, (2) Multilevel regression models provide interpretable effect estimates while accounting for clustering within samples and distinguishing within-sample from between-sample associations, and (3) Random-effects meta-analysis of sample-specific slopes quantifies cross-context consistency and heterogeneity. Convergent findings across all three approaches strengthen confidence that a risk factor is robust rather than an artifact of a single modeling choice.

### Conditional random forest analysis

We first used conditional random forests (CRFs) to place all risk factors in direct multivariate competition and identify those that most strongly improved the model’s ability to account for radical intentions. Model performance was assessed using out-of-bag (OOB) estimation, a built-in cross-validation procedure that tests the model on observations not used during its estimation. Out-of-bag (OOB) performance was comparable across raw, grand-mean centered, and within-sample centered specifications (Table 3), indicating that conclusions were not sensitive to centering choices.

**TABLE 3:** Out-of-bag performance metrics for the conditional random forests.

Specification	OOB MSE	OOB MAE
Centered within countries	1.434	0.763
Grand-mean centered	1.373	0.728
Raw	1.368	0.728

**FIGURE 1:**
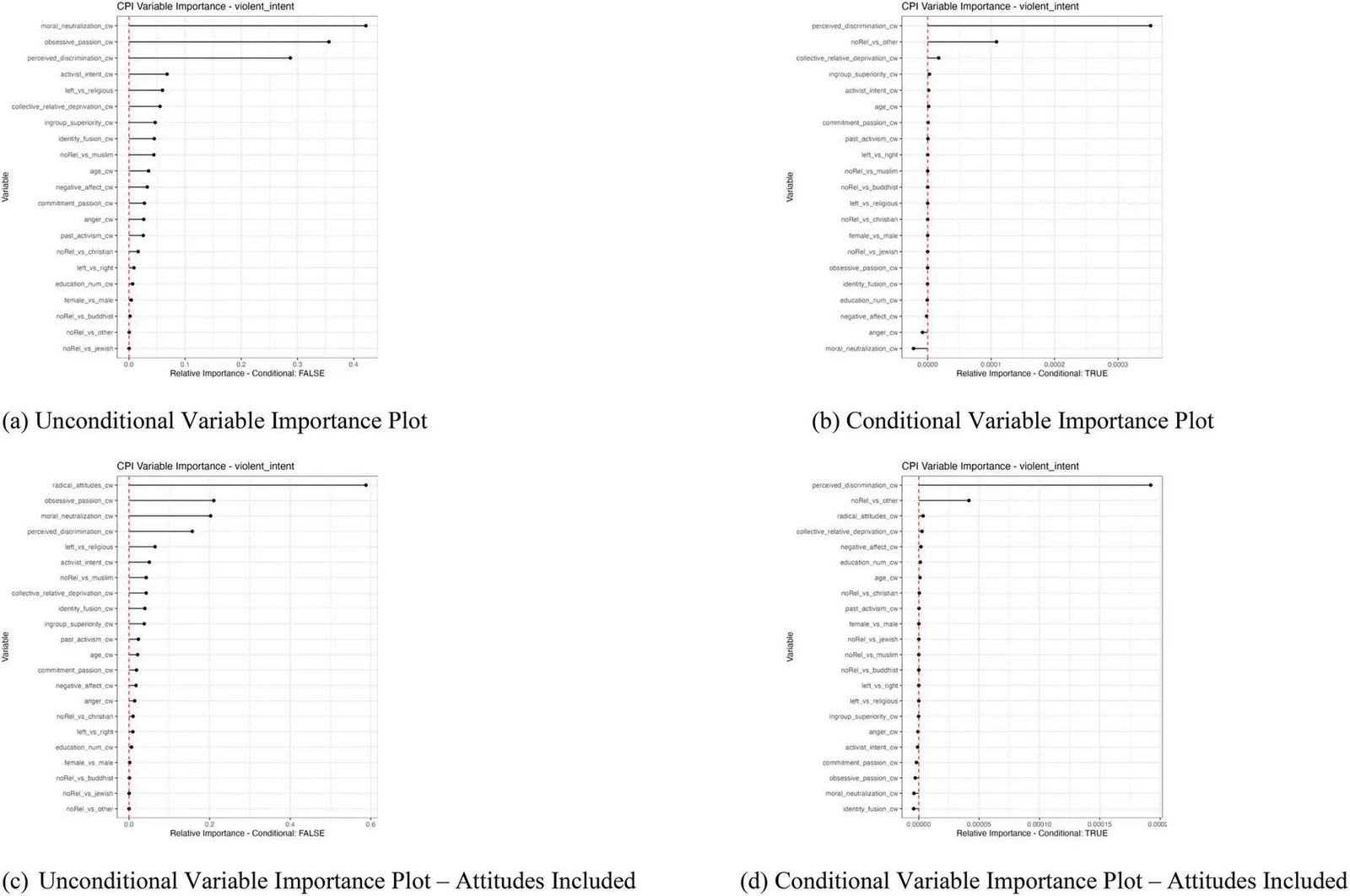
Unconditional and conditional variable importance plots for the random forest analysis. **(a)** Unconditional variable importance plot. **(b)** Conditional variable importance plot. **(c)** Unconditional variable importance plot—attitudes included. **(d)** Conditional variable importance plot—attitudes included.

**FIGURE 2:**
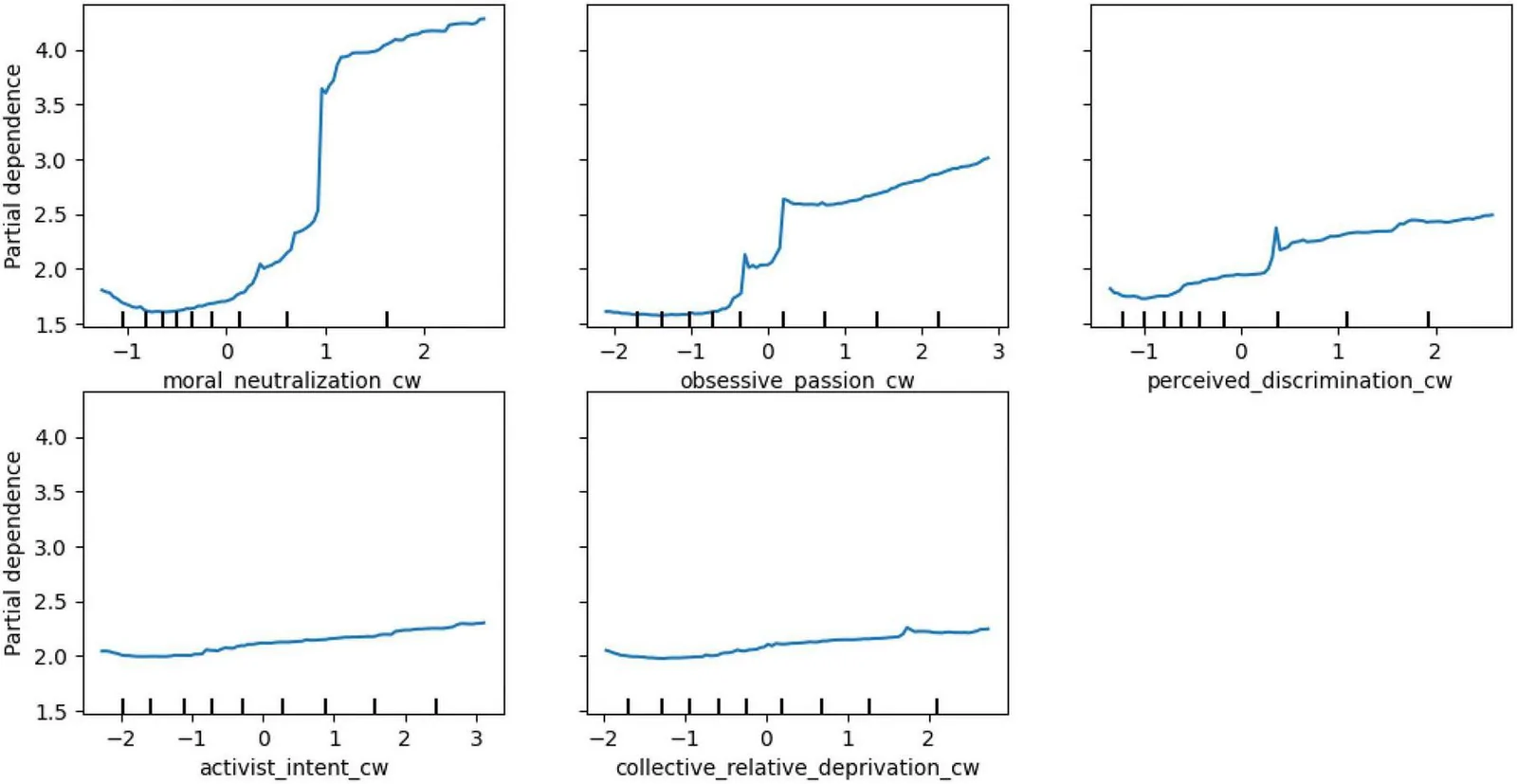
Partial dependency plots for largest risk factors in random forest analysis.

Across specifications, unconditional variable-importance plots (Figure 1a and Table 4) identified obsessive passion, moral neutralization, and perceived discrimination as the three most important risk factors. To interpret the direction and functional form of these associations, we examined partial dependence plots (Figure 2). Radical intentions increased approximately linearly with perceived discrimination, whereas increases in moral neutralization and obsessive passion were especially pronounced at above-average levels (i.e., values above 0 after standardization).

**TABLE 4:** Average permutation importance for theoretically central risk factors across random-forest specifications.

Condition-ality	Specification	Risk factors	Mean importance
Conditional = False	Raw	Moral neutralization	0.411
Conditional = False	Raw	Obsessive passion	0.393
Conditional = False	Raw	Perceived discrimination	0.282
Conditional = True	Raw	Moral neutralization	0.000 ^a^
Conditional = True	Raw	Obsessive passion	0.000 ^a^
Conditional = True	Raw	Perceived discrimination	0.000 ^a^

*^a^*See Figure 1b for comparison.

Conditional importance analyses, which estimate each variable’s unique contribution when correlated risk factors are taken into account (Figure 1b), again highlighted the same three risk factors but elevated perceived discrimination as the most distinctive risk factor. Finally, when including radical attitudes in the preregistered specification, radical attitudes unsurprisingly showed high unconditional importance (Figure 1c); however, the conditional importance ranking again placed perceived discrimination among the most distinctive risk factors (Figure 1d). Together, the machine-learning results converged on a common set of risk factors—obsessive passion, moral neutralization, and perceived discrimination—that provided nonredundant associations across specifications.^2^

### Multilevel regression models

Next, we estimated multilevel regression models to translate the machine-learning rankings into interpretable effect estimates while accounting for the nesting of respondents within samples. Risk factors were decomposed into within-sample and between-sample components to distinguish whether associations reflected differences between individuals within the same context (within-sample) versus differences between contexts in average levels (between-sample).

Introducing random slopes for the three focal risk factors (moral neutralization, obsessive passion, perceived discrimination) substantially improved model fit relative to a random-intercept-only specification (χ^2^ = 320.11, *df* = 9, *p* < 0.001); we therefore interpret the random-slopes model as the primary inferential model. Fixed-effect estimates (Table 5) indicated that moral neutralization (*b* = 0.42, *t* = 14.67), obsessive passion (*b* = 0.29, *t* = 12.24), and perceived discrimination (*b* = 0.24, *t* = 7.87) showed the largest within-sample effects.

**TABLE 5:** Fixed-effects estimates for key risk factors of radical intentions in the random-slopes multilevel model.

Level	Risk factor	Estimate	Std. Error	*t*-value	CI
Within countries	Moral neutralization	0.42	0.03	14.67	95% CI [0.37, 0.48]
Within countries	Perceived discrimination	0.24	0.03	7.87	95% CI [0.18, 0.30]
Within countries	Obsessive passion	0.29	0.02	12.24	95% CI [0.25, 0.34]
Between countries	Moral neutralization	0.39	0.24	1.59	95% CI [-0.09, 0.87]
Between countries	Perceived discrimination	-0.14	0.14	-1.03	95% CI [-0.42, 0.13]
Between countries	Obsessive passion	0.27	0.20	1.33	95% CI [-0.13, 0.66]

Between-sample associations for these risk factors were weaker and less precisely estimated (Table 5). This pattern implies that the strongest signal lies in differences between individuals within the same context: individuals higher on moral neutralization, obsessive passion, and perceived discrimination than others in their sample reported higher radical intentions. A model restricted to these three focal risk factors yielded nearly identical within-sample slopes—obsessive passion (*b* = 0.31, *SE* = 0.02, *t* = 14.42), moral neutralization (*b* = 0.45, *SE* = 0.03, *t* = 15.84), and perceived discrimination (*b* = 0.28, *SE* = 0.03, *t* = 8.95)—indicating that the focal associations did not hinge on auxiliary covariates.

### Meta-analytic synthesis across samples

**FIGURE 3:**
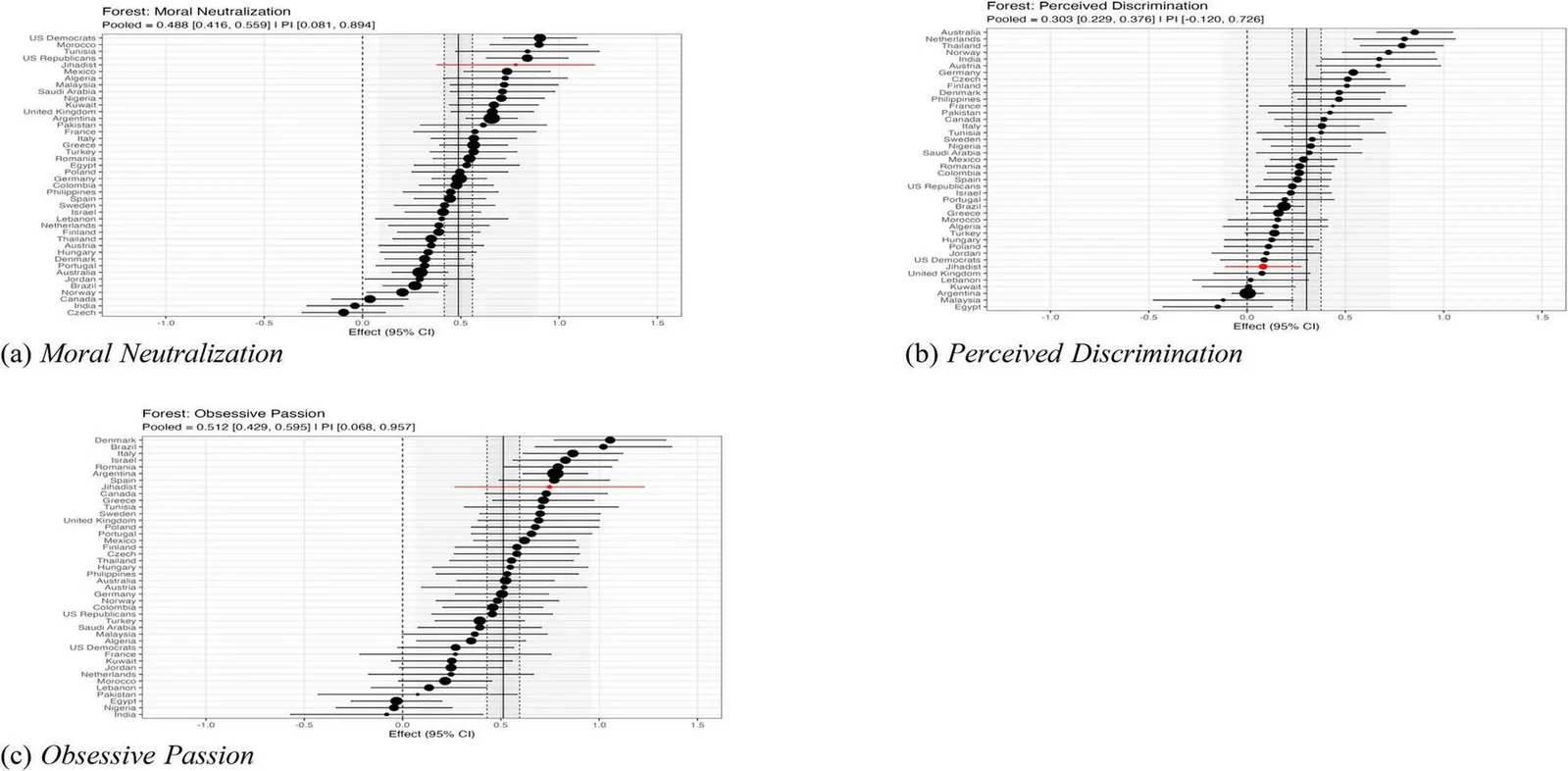
Forest plots for the focal risk factors. **(a)** Moral neutralization. **(b)** Perceived discrimination. **(c)** Obsessive passion.

Finally, we made cross-context variability explicit by estimating regression slopes separately within each sample and pooling them using random-effects meta-analysis. This approach yields an average association while also quantifying heterogeneity (i.e., the extent to which effect sizes vary across contexts). Across the 41 samples, obsessive passion, moral neutralization, and perceived discrimination again produced the largest pooled effects (Table 6), with average slopes ranging from 0.51 to 0.30. Forest plots (Figure 3) showed that these associations were consistently positive across most contexts, although their magnitudes varied (see Table 6). Sensitivity analyses examining small-sample asymmetry did not materially alter the pooled estimates (e.g., trim-and-fill and contour-enhanced funnel plots; Supplementary Figure 4; also see Duval and Tweedie, 2000; Hilgard, 2016; Peters et al., 2008).

**TABLE 6:** Random-effects meta-analysis of country-specific slopes for key risk factors of radical intentions.

Risk factor	Estimate	95% CI (lower)	95% CI (upper)	*I* ^2^	τ ^2^
Moral neutralization	0.49	0.42	0.56	78.1	0.04
Perceived discrimination	0.30	0.23	0.38	81.8	0.05
Obsessive passion	0.51	0.43	0.60	69.1	0.05

Other risk factors showed smaller and less consistent pooled effects when considered alongside all candidate risk factors (see Supplementary Figure 1). Overall, the convergent evidence from CRFs, multilevel models, and meta-analysis identifies obsessive passion, moral neutralization, and perceived discrimination as the most robust risk factors of radical intentions across diverse contexts.

### Risk factors of radical intentions in the incarcerated jihadist sample

We next tested whether the cross-national focal risk factors were also correlated with radical intentions in an incarcerated sample of jihadist offenders in Spain (*N* = 80). Given the small sample size, we used two complementary analyses that parallel the cross-national triangulation: (1) a penalized-regression tournament (LASSO) in which risk factors compete under regularization (analogous to the cross-national random-forest selection), and (2) a benchmarking analysis comparing focal risk-factor slopes to the pooled cross-national estimates (analogous to the cross-context synthesis).

### Penalized regression (LASSO)

The cleaned jihadist dataset contained 14 risk factors that overlapped with the broader project (i.e., 12 psychological variables, plus education and age as demographic variables). To reduce overfitting in this small-*N* setting, we fit LASSO models with five-fold cross-validation repeated 20 times (100 resamples total). Median imputation and standardization were performed within each resample so that all risk factors competed on a common scale during penalty tuning.

Zero-order correlations (Supplementary Figure 5) suggested that moral neutralization and obsessive passion were positively associated with radical intentions in this sample, whereas perceived discrimination showed a weaker association. Cross-validated performance across the penalty grid is shown in Supplementary Figure 6. At the selected penalty, the retained standardized coefficients (Supplementary Figure 7 and Table 7) again highlighted obsessive passion and moral neutralization as the largest positive risk factors, whereas the coefficient for perceived discrimination was small.

**TABLE 7:** Active LASSO coefficients.

Rank	Risk factor	Coefficient	Direction
1	Obsessive passion	0.590	Risk factor
2	Moral neutralization	0.580	Risk factor
3	Past activism	-0.270	Protective factor
4	Ingroup superiority	0.269	Risk factor
5	Identity fusion	0.255	Risk factor
6	Education num	-0.220	Protective factor
7	Commitment passion	-0.103	Protective factor
8	Anger	-0.086	Protective factor
9	Negative affect	-0.068	Protective factor
10	Age	0.063	Risk factor
11	Collective relative deprivation	-0.029	Protective factor
12	Perceived discrimination	0.017	Risk factor

Bootstrap inclusion probabilities (Supplementary Figure 8) indicated that moral neutralization (99%) and obsessive passion (97%) were among the most stable signals in the penalized model. Several additional risk factors showed moderate inclusion probabilities (e.g., past activism, education, identity fusion, in-group superiority) but given the small sample size these exploratory signals should be interpreted cautiously.

### Benchmarking focal risk factors against cross-national estimates

To benchmark the incarcerated-sample associations against the broader cross-national evidence, we estimated standardized regression coefficients for each focal risk factor in the jihadist sample and compared them to the pooled cross-national estimates. Figure 3 overlays the jihadist estimates (in red) on the cross-national forest plots.

Consistent with the cross-national findings, moral neutralization (*b* = 0.98, 95% CI [0.68, 1.28]) and obsessive passion (*b* = 0.68, 95% CI [0.49, 0.88]) were clearly positive correlates of radical intentions. Perceived discrimination was directionally consistent but weaker and more uncertain (*b* = 0.16, 95% CI [-0.01, 0.32]). Thus, two of the three cross-national risk factors—moral neutralization and obsessive passion—showed convergent positive associations in the incarcerated extremist sample, whereas perceived discrimination showed attenuation and greater uncertainty.

Taken together, the cross-national and incarcerated-sample estimates suggest that part of the general-population picture was echoed in the jihadist detainee sample: moral neutralization and obsessive passion remained positive correlates of radical intentions, whereas perceived discrimination—while pointing in the same direction—carried wider uncertainty bands.

## Discussion

The present research is an extensive cross-national test of risk factors of radical intentions—the willingness to use violence to advance an ideology (Wolfowicz et al., 2021). Drawing on 42 samples (41 general population samples and one sample of incarcerated extremists) from 40 countries spanning six continents and over 10,000 participants, this study combined complementary analytic approaches—conditional random forests, multilevel modeling, and random-effects meta-analysis—to identify the most robust correlates of radical intentions across ideological and cultural boundaries.

Building on the foundational meta-analysis provided by Wolfowicz et al. (2021), which narrowed over a hundred potential correlates to the 12 most empirically robust risk factors, the present multivariate competition reveals three variables that emerge as the most robust independent correlates of violent intentions, forming a moral–motivational–grievance combination: moral neutralization, obsessive passion, and perceived discrimination.

These findings reveal a psychologically coherent set of risk factors that showed broadly similar associations across the ideological domains assessed here, thereby refining the empirical foundation laid by Wolfowicz et al. (2021) into a more parsimonious set of core correlates. Across diverse national contexts and ideological groups, higher levels of moral neutralization, obsessive passion, and perceived discrimination were consistently associated with stronger willingness to use violence in support of a cause. This moral–motivational–grievance combination was associated with radical intentions across contexts, while not determining how much ideology-specific content shapes entry into these processes or what links violent intentions to actual violent acts. This combination held robustly in general-population samples across political and religious ideologies, and was partially replicated among incarcerated jihadists, where moral neutralization and obsessive passion remained robust risk factors while perceived discrimination showed a less consistent association. Collectively, the results indicate that the willingness to use violence in support of a cause is associated with a set of cross-culturally consistent psychological processes — how individuals morally justify harm, commit to a cause, and perceive their group’s treatment — and that these associations hold similarly across left-wing, right-wing, and religious contexts. Notably, indicators of which ideological group an individual supported added little predictive value once these three risk factors were taken into account (see text footnote 2), suggesting that what most clearly distinguishes individuals higher in radical intentions is less which cause they hold than their standing on these moral, motivational, and grievance-related dimensions. Because the present analyses are cross-sectional and correlational, however, they do not permit causal or directional inference; we therefore describe these three risk factors in associational terms throughout this Discussion, and references to underlying “processes” denote theorized rather than demonstrated pathways. The consistency of these associations across analytic methods, national contexts, and ideological domains nonetheless provides a strong basis for theory refinement and for generating causal hypotheses to be tested in future longitudinal and experimental work.

### Robust risk factors associated with radical intentions

The consistent emergence of moral neutralization, obsessive passion, and perceived discrimination points to a shared set of psychological correlates associated with radical intentions, one that recurs with striking similarity across diverse ideological and cultural settings. In the sections that follow, we consider independently each of these three risk factors and its implications for longstanding theories of radicalization.

### Moral neutralization: bypassing ethical constraints

Across analytic methods, moral neutralization consistently emerged as one of the most robust and stable risk factors of radical intentions. Participants who endorsed cognitive justifications for violence—such as viewing aggression as acceptable in the service of a cause—reported markedly higher intentions to engage in ideological violence. This association aligns with Bandura’s (1999) theory of moral disengagement, which holds that individuals can temporarily suspend moral self-regulation by justifying or minimizing the immorality of harmful actions. Decades of research, including analyses of interviews and autobiographical accounts of terrorists, similarly indicates that the belief that virtually any means, including violence, are justified in pursuit of a righteous cause lies at the core of the terrorist mentality (Borum, 2011; Doosje et al., 2016; Horgan, 2005). Across ideological contexts—from revolutionary Marxism to jihadism—violent actors frequently argue that violence represents the most efficient path toward a morally good society, thereby legitimizing its use (Rapoport, 2013). When radical means are construed as serving a greater moral good in the long run, despite immediate harm, they become morally permissible (Snook et al., 2025). Taken together, these findings point to a coherent moral calculus in which the perceived righteousness of one’s cause outweighs the moral costs of the violence required to advance it. Consistent with this account, recent evidence indicates that comparable disinhibitory processes are present in real-world cases of radicalization (Pfundmair et al., 2026).

To the extent that perceived discrimination is associated with greater motivation to act, moral reasoning—or its suspension—may be what reframes grievance as permission for aggression, often through processes such as dehumanization that strip targets of moral worth and attenuate empathic concern (Haslam and Loughnan, 2014). We emphasize that these proposed pathways are conjectural. The present analyses are correlational and cross-sectional, and the multivariate and nonlinear patterns we observe are equally compatible with reverse-ordered or reciprocal relationships (e.g., radical intentions increasing moral neutralization) and with unmeasured common causes. Establishing temporal precedence and ruling out alternative explanations will require longitudinal and experimental designs.

Importantly, this effect appears to operate with comparable strength across ideological contexts, suggesting a widely shared psychological process rather than an ideology-specific phenomenon. Whether framed as defending faith, protecting one’s group, or advancing justice, moral neutralization is associated with viewing violence as morally permissible rather than immoral.

Further illustrating this process, the partial-dependence curves—which graphically illustrate the marginal effect of a single risk factor (moral neutralization) on radical intentions while averaging over the distribution of all other risk factors— were threshold-like in form: estimated radical intentions were relatively flat across lower values of moral neutralization and substantially higher above a certain level. Descriptively, this nonlinear shape is consistent with a ‘tipping point’ dynamic, in which radical intentions are markedly higher only once moral neutralization is high. Because the data are cross-sectional, this shape describes how intentions covary with moral neutralization across individuals; it does not establish that moral neutralization develops, ‘solidifies,’ or escalates within individuals over time. With that caveat, the nonlinearity may nonetheless be relevant to prevention, suggesting potential value in addressing moral justifications for violence before they reach the levels associated with sharply elevated radical intentions.

### Obsessive passion: a consuming ideological commitment

The second pillar of this combination, obsessive passion, indexes the motivational dimension of radical intentions in our data. According to the Dualistic Model of Passion (Vallerand et al., 2003; Vallerand, 2015), passion toward an activity or cause can take two distinct forms. Harmonious passion reflects voluntary, flexible engagement: individuals choose to invest in a cause they value while maintaining balance with other life goals. Obsessive passion, by contrast, reflects rigid, compulsive engagement: the cause becomes ego-involving and difficult to disengage from, compelling behavior even when it conflicts with other values or personal well-being (Bélanger, 2021).

Our results provide strong empirical support for the theory of passion in the context of ideology: individuals scoring high on obsessive passion were significantly more likely to endorse violent means for an ideology. This relationship was robust across cultures and persisted even after controlling for moral cognition, emotional variables, and demographics. Partial-dependence patterns suggested that increases in violent intentions were especially pronounced at above-average levels of obsessive passion, consistent with the possibility that highly ego-involving investment in a cause is particularly consequential. Importantly, harmonious passion—measured as a control variable—did not emerge as a stable correlate of radical intentions, confirming the theoretical distinction between adaptive and maladaptive passion.

Conceptually, the Dualistic Model frames obsessive passion as a form of engagement in which ideology functions less as a belief system than as an identity-regulating mechanism. On this account, when personal worth is fused with a cause, challenges to the cause may be experienced as existential threats (Bélanger et al., 2021; Rip et al., 2012), and violence may take on identity-relevant rather than purely instrumental meaning. Our cross-sectional data are consistent with this account—obsessive passion was among the most robust correlates of radical intentions—but cannot confirm that obsessive passion produces these shifts. The finding that obsessive passion is associated with radical intentions equally in secular and religious contexts underscores its psychological generality: the dynamics of overcommitment, ego-investment, and loss of self-regulatory control transcend ideological content.

### Perceived discrimination: experiencing collective grievance

The third key risk factor, perceived discrimination, captures the subjective sense that one’s group is unfairly treated or excluded. It is the most explicitly social and intergroup component of this combination, locating radical intentions within perceptions of collective injustice rather than purely individual cognition or motivation. Such perceptions have been theorized to contribute to collective resentment and retaliatory action (Doosje et al., 2013). In our data, perceived discrimination showed a near-linear association with radical intentions: across individuals, higher perceived discrimination was accompanied by higher willingness to use violence in support of an ideological cause. Unlike moral neutralization and obsessive passion, this association showed no clear threshold; instead, increases in perceived discrimination corresponded to approximately proportional increases in radical intentions.

Although smaller in magnitude than moral neutralization and obsessive passion (average slope ≈ 0.30), perceived discrimination showed a unique association with radical intentions in conditional analyses. Even when moral and motivational variables were controlled, perceived discrimination remained independently associated with radical intentions, suggesting that perceived injustice is not merely reducible to those other risk factors. This asymmetry is theoretically meaningful: large segments of the population perceive their group as unfairly treated, yet only a small minority endorse or enact violence on behalf of an ideological cause (Horgan, 2005; McCauley and Moskalenko, 2008, 2017; Kruglanski et al., 2019; Rottweiler and Gill, 2022). Thus, perceived discrimination appears to be a relevant grievance-related correlate of radical intentions, though its association was less consistent than those observed for the moral and motivational components of this combination. In the incarcerated jihadist sample, perceived discrimination was also less robustly associated with radical intentions than in the general-population samples; however, because this is a between-sample comparison rather than a within-person trajectory, the data cannot show that perceived discrimination matters less “later” in radicalization.

Taken together, these findings position perceived discrimination as the grievance-related component of this combination: independently associated with radical intentions, but more context-dependent than moral neutralization and obsessive passion. This finding reinforces work linking collective relative deprivation to intergroup hostility (Gurr, 1970; Obaidi et al., 2019) and is consistent with gateway models of radicalization, in which perceived injustice is theorized to contribute to initial mobilization, while additional moral and motivational factors may help distinguish violent from nonviolent forms of engagement (Guimond et al., 2025). The cross-sectional design, however, cannot establish that these factors operate in sequence, that grievance is transformed into violent readiness, or that any one factor is a prerequisite for another.

### Theoretical implications

Having identified this reduced set of risk factors through direct empirical competition among the 12 strongest correlates cataloged by Wolfowicz et al. (2021), the present findings provide a clear lens for refining and integrating longstanding theoretical frameworks of radicalization.

While the 3N model (Kruglanski et al., 2019) emphasizes Need, Narrative, and Network as the core components of radicalization, the present findings show how these abstract elements are instantiated in measurable psychological constructs. Specifically, obsessive passion can be interpreted as the motivational Need for personal significance and self-validation; moral neutralization corresponds to the violence-justifying Narrative; and perceived discrimination maps onto both the arousal of Need—through feelings of humiliation or injustice—and the grievance that is amplified and collectivized through the Network. This dynamic reflects a key feature of the 3N model: radical groups transform private grievances into shared, ideologically weaponized narratives of victimhood. These processes delineate the micro-level risk factors that operationalize the 3N structure, with perceived discrimination linking Need arousal and Network-level social validation. We note, however, that our data speak to individual-level correlates of radical intentions and not to the group-level processes these models invoke—such as the social dynamics through which networks promote and sustain radical narratives—which lie beyond the scope of the present design.

Building on this foundation, this combination also aligns closely with Moghaddam’s (2005) staircase model of terrorism, which depicts radicalization as a gradual ascent from perceived injustice on the “ground floor” to moral justification and violent action at the “top floor.” This set of correlates mirrors this progression: perceived discrimination corresponds to the sense of injustice and blocked mobility at the base; obsessive passion reflects the motivational escalation and narrowing of identity that accompany the upward progression; and moral neutralization captures the cognitive transformation that enables moral disengagement and violence at the upper levels. In this way, these three risk factors are consistent with the constructs Moghaddam’s model invokes at successive steps, though our cross-sectional data speak to the co-occurrence of these constructs rather than to the temporal transitions the model describes; we cannot show that structural grievance evolves into personalized commitment and moral justification.

These continuities also extend to Borum’s (2011) four-stage model, which outlines a cognitive progression from grievance (“It’s not right”) to perceived injustice (“It’s not fair”), to attribution of blame (“It’s your fault”), and finally to moral justification (“You are evil, and violence is justified”). This set of correlates offers a streamlined analog of this sequence: perceived discrimination corresponds to the initial grievance and unfairness appraisals; obsessive passion parallels the emotional intensification and identity commitment that the model situates ahead of action; and moral neutralization corresponds to its final stage, in which violence is morally justified—though, as with the other stage models, our cross-sectional data index the co-occurrence of these constructs rather than a within-person progression through Borum’s stages.

From a broader sociological perspective, these correlates also connect to relative deprivation and social movement theories (Gurr, 1970; Della Porta, 1995), which emphasize that perceptions of inequality and exclusion—rather than absolute deprivation—are associated with collective mobilization. Perceived discrimination in our findings captures precisely this subjective sense of injustice, while obsessive passion and moral neutralization distinguish the minority who endorse violence from the majority who, despite similar grievance, do not. In this way, these correlates bridge macro-level structural explanations of protest with micro-level psychological risk factors of radicalization, offering a unifying lens on how social discontent is linked to ideological violence.

Compared to prior meta-analytic work, the current study also clarifies the hierarchy among competing psychological risk factors. When modeled simultaneously in direct competition, variables such as identity fusion, in-group superiority, and anger—previously considered central correlates of radical intentions—showed attenuated associations beyond these three risk factors. This attenuation underscores how the meta-analytic shortlist, while comprehensive, contained considerable overlap; the current tournament-style approach distilled the field further, identifying them as the risk factors that most consistently retained independent, non-redundant associations across analytic approaches—consistent with the goal of advancing from broad synthesis toward theoretical parsimony. Of particular interest is identity fusion, which exhibited the strongest pooled-correlation with radical intentions in Wolfowicz et al.’s (2021) meta-analysis and is unique in explicitly merging personal and social identity into a visceral sense of “oneness” with the collective (Swann et al., 2012; Gómez et al., 2020). Despite this strong meta-analytic support and theoretical prominence, the present results indicate that obsessive passion and moral neutralization emerged as stronger proximal risk factors than identity fusion itself. Importantly, most prior research on fusion has focused mainly on fusion with concrete social groups or leaders rather than with abstract values or ideologies (Swann et al., 2012; Feigin et al., 2026). Consequently, the current findings—obtained largely in ideology-driven contexts—suggest that this set of correlates may be especially well-suited to radicalization rooted in commitment to a political or religious cause, whereas group- or leader-centered pathways may rely more heavily on fusion processes. This relative importance does not exclude upstream or indirect effects. For instance, fusion may heighten vulnerability to obsessive passion, which in turn relates to violent readiness, while anger may arise from perceived discrimination but contribute less once moral justification is accounted for. Future research should examine these potential upstream and indirect pathways across ideology-driven versus group-centered contexts of radicalization. The comparatively limited role of identity fusion here may also partly reflect our predominantly general-population samples, in which fusion with a specific group or leader is typically less salient than in the smaller, already-radicalized populations where strong fusion effects are usually observed.

By triangulating results from machine learning, multilevel modeling, and meta-analytic synthesis, this study provides rare methodological confirmation that the identified effects are robust across analytic paradigms. The convergence of machine-learning, inferential, and integrative evidence underscores the reliability of this set of correlates. More broadly, this convergence consolidates and connects multiple lines of radicalization research—motivational (3N), cognitive (Staircase and Borum), and sociological (relative deprivation and social movement theory)—into a unified, empirically substantiated account of how moral, motivational, and perceptual risk factors are jointly associated with radical intentions.

### Practical implications

The consistency of these three risk factors across methods, ideologies, and cultures yields suggestive directions for prevention and intervention. Because our data are correlational, the following implications are necessarily provisional: they assume that these correlates play some causal role, an assumption that intervention research—not the present study—must establish. One key implication concerns the need to target moral neutralization directly. Intervention research could test whether reducing moral justifications for violence lowers radical intentions. Cognitive-behavioral and dialogical interventions that help individuals recognize inconsistencies in their moral reasoning—such as equating ideological violence with self-defense—may help restore moral self-regulation and empathy. Educational initiatives emphasizing universal ethical principles may further reduce susceptibility to ideologically framed exceptions and re-establish shared moral boundaries.

A second implication involves promoting harmonious rather than obsessive passion. The Dualistic Model of Passion (Vallerand et al., 2003) provides a clear framework for intervention, emphasizing that the goal is not to suppress passion for causes but to achieve balance. Programs that nurture autonomy, self-complexity, and emotional regulation may be promising candidates for fostering more balanced engagement with ideological causes. For example, techniques such as self-reflection, mindfulness, and identity diversification may prevent ideological commitment from becoming ego-absorptive and compulsive. Moreover, the use of implementation intentions (Gollwitzer, 1999)—specific if–then self-regulation plans (e.g., “If I feel compelled to act impulsively for my cause, then I will take ten minutes to reflect before responding”)—has been shown to help individuals anticipate and regulate passion-driven impulses in ways that foster more balanced, harmonious engagement while reducing rigid, obsessive forms of commitment (Bélanger et al., 2019b). At the community level, fostering balanced civic engagement—through volunteering, peaceful activism, and dialogue—may channel political or ideological energy constructively rather than destructively.

A third practical avenue concerns perceived discrimination and grievance narratives. Because perceived discrimination was robustly associated with radical intentions, prevention efforts may benefit from addressing both actual unfair treatment and the interpretive frames through which grievances are understood. Institutional policies that promote inclusion, transparency, and equitable treatment remain theoretically relevant, although the present data cannot evaluate their causal effects on radical intentions. At the same time, communication around such policies should avoid unintentionally reinforcing overgeneralized grievance narratives or perceptions of pervasive exclusion, which extremist movements may exploit (Wilkes and Wu, 2019). Community outreach and strategic communication may therefore be most useful when they combine credible acknowledgment of real discrimination, including anti-Muslim prejudice and related forms of exclusion (Imhoff and Recker, 2012), with efforts to counter conspiratorial, absolutist, or violence-justifying interpretations of grievance.

Finally, the cross-ideological consistency of these three risk factors underscores the value of broadly applicable rather than strictly ideology-specific prevention strategies. Indeed, because moral neutralization, obsessive passion, and perceived discrimination are associated with radical intentions across political, religious, and cultural divides, counter-radicalization efforts that target these shared psychological risk factors, rather than ideological content alone, may generalize across contexts more readily than ideology-specific approaches; whether such an approach is also more effective is a question only intervention research can answer. Prior intervention research is at least consistent with this direction: studies of preventing and countering violent extremism (PCVE) report that interventions aimed at multiple groups or the general population outperform those focused on a single ideological community, in part because they avoid the stigmatization and profiling that can exacerbate perceived marginalization (Jugl et al., 2021).

Evidence further suggests that multicomponent interventions have a compounding advantage over single-factor approaches (Webber et al., 2018; Weisburd et al., 2022). Accordingly, PCVE initiatives might consider jointly addressing obsessive passion, moral neutralization, and perceived discrimination—for example by reducing sources of perceived deprivation while fostering norms of nonviolence, trust, shared civic identity, and mutual assistance (Bélanger, 2017; Weisburd et al., 2022)—though the value of doing so would need to be confirmed in intervention trials. Together, these strategies may help reduce ingroup–outgroup divisions and weaken the psychological processes associated with radical intentions.

### Limitations

Several limitations warrant consideration. The cross-sectional design precludes causal inferences regarding the direction of effects: it remains unclear whether moral neutralization, obsessive passion, and perceived discrimination lead to radical intentions or vice versa. Longitudinal and experimental research is therefore essential to establish temporal precedence and causality. Additionally, the radical intentions variable showed positive skewness, particularly in the jihadist sample, with few participants endorsing high levels of violent intentions. While this distribution is expected in general-population samples, the restricted variance likely underestimates effect sizes. The study also included only one sample of incarcerated extremists, and larger and more diverse samples of both active and former extremists are needed to properly assess the generalizability of these correlates to individuals with direct involvement in ideological violence. Findings from the incarcerated sample, in particular, should be regarded as preliminary: given its small size (*N* = 80), the width of the associated confidence intervals, the instability of penalized estimates in small samples, and its restriction to Spanish jihadist offenders, these associations require replication in larger and more diverse samples of individuals who have engaged in ideological violence. Relatedly, because the radical-intentions items administered in this sample referenced the cause of Islam specifically, our benchmarking comparisons index intentions toward the same ideological cause associated with this sample’s documented offending, which strengthens the interpretability of the comparison; at the same time, this ideology-specific wording means these findings speak to generalization within a jihadist frame of reference and cannot establish whether these correlates extend to violent intentions organized around other causes.

A further limitation concerns the interpretation of our pooled estimates given substantial between-sample heterogeneity (see Table 6). Because I^2^ is a relative index that inflates when the contributing samples are large and individually precise (Borenstein et al., 2017; Rücker et al., 2008), these values primarily indicate that genuine cross-context variation exists rather than that the pooled estimate is uninformative; the absolute dispersion was in fact modest. The 95% prediction intervals in Figure 3 make this concrete (IntHout et al., 2016): they lie entirely above zero for obsessive passion and moral neutralization but include zero for perceived discrimination, consistent with our reading of perceived discrimination as the smallest and most context-dependent estimate among the three focal correlates. We therefore caution against treating the pooled slopes as universal effect sizes—in any given context the true association may be appreciably stronger or weaker—particularly because samples differ in the focal in-group to which the ideology-tailored items referred (a governing party, Islam, or Hinduism). Notably, our conclusions do not depend on these magnitudes: the conditional random forests and a multilevel model whose random slopes explicitly accommodate cross-context variation (see Table 5) converge on the same three risk factors. We thus regard the reproducible direction and rank-ordering of these three risk factors, rather than the exact magnitude of any pooled coefficient, as a robust contribution, and we identify the national-level moderators discussed below as a promising route to explaining the observed heterogeneity.

The operationalization of ideology—based on the dominant political or religious affiliation per country—simplifies the multidimensional nature of ideological identification. Future work should include finer-grained ideological measures and test for within-ideology variance. Finally, despite inclusion of non-Western contexts, data collection via online panels may underrepresent marginalized or offline populations most vulnerable to recruitment. Thus, although cross-cultural generality is supported by the data, representativeness remains imperfect.

Relatedly, because recruitment restricted participation in each context to self-identified supporters or adherents of the focal group (the governing political party, or Islam and Hinduism where applicable), our samples were drawn from politically or religiously dominant groups rather than from populations more typically characterized by grievance, marginalization, or collective disadvantage. This distinguishes our samples from many real-world populations at elevated risk of radicalization, whose radical intentions may be embedded in experiences of structural exclusion and discrimination (e.g., Obaidi et al., 2019). Whether the correlates identified here generalize to members of marginalized or minority groups—for whom perceived discrimination, in particular, may operate differently—remains an open question for future research.

### Future directions

Future research should pursue causal and dynamic modeling of this set of correlates. Experimental manipulations of moral justification, obsessive passion, or perceived discrimination could clarify causal ordering. Longitudinal studies could test whether obsessive passion and moral neutralization increase radical intentions over time or mediate the effect of perceived discrimination. Cross-lagged panel models could identify bidirectional feedback loops—for example, whether obsessive passion leads to stronger moral neutralization, or vice versa.

A second direction involves enriching the existing dataset with macro-level indicators to capture the broader structural and economic contexts within which radical intentions develop. The present study’s multilevel design already differentiates between within- and between-country variance, but future iterations could explicitly model cross-level interactions between individual psychological risk factors and national-level conditions. For example, integrating Gross Domestic Product (GDP), the Gini index of income inequality, and standardized governance or corruption indices could help assess whether socioeconomic opportunity, inequality, and institutional trust moderate the strength of these associations.

Such integration would enable tests of theoretically meaningful hypotheses—for instance, whether perceived discrimination increases radical intentions more strongly in countries with high inequality, or whether moral neutralization exerts greater influence in contexts with weak governance and low institutional legitimacy. Including macro-level covariates would also illuminate whether obsessive passion flourishes under conditions of collective instability or restricted civic participation.

By combining psychological micro-dynamics with societal macro-structures, future work could yield a truly comprehensive understanding of radicalization as a multilevel phenomenon—one shaped not only by individual cognition and emotion but also by the socioeconomic and political environments that make certain psychological pathways more likely to emerge. This cross-level enrichment would enable testing of how personal vulnerabilities and structural contexts interact to drive extremist commitment.

## Conclusion

Across countries and ideologies, this study identified a robust, cross-culturally consistent set of correlates associated with radical intentions: moral neutralization, obsessive passion, and perceived discrimination. Advancing Wolfowicz et al. (2021) through multivariate adjudication, the study shows that these three factors most consistently retained independent associations with radical intentions across machine-learning, multilevel, and meta-analytic tests. In the exploratory incarcerated jihadist sample, moral neutralization and obsessive passion showed convergent positive associations, whereas perceived discrimination was directionally consistent but attenuated. Future intervention studies could test whether addressing these three factors reduces radical intentions by reinforcing moral constraints against violence, promoting harmonious rather than obsessive ideological engagement, and addressing real and perceived discrimination. Together, they offer a parsimonious set of candidate targets for future prevention and intervention research.

## Data availability statement

The raw data supporting the conclusions of this article will be made available by the authors, without undue reservation.

## Ethics statement 

The studies involving humans were approved by the Institutional Review Boards of New York University Abu Dhabi and Universidad Nacional de Educación a Distancia. The studies were conducted in accordance with the local legislation and institutional requirements. The participants provided their written informed consent to participate in these studies.

## Footnotes

1 We note that ‘activism’ here follows the activism–radicalism distinction (Moskalenko and McCauley, 2009): it denotes legal, non-violent political engagement, conceptually separate from the violent behavioral engagement that radical intentions concern, and is included only as one of Wolfowicz et al.’s candidate correlates.

2 Group differences (e.g., political vs. religious, left vs. right) were included in all models but consistently ranked near zero in both unconditional and conditional importance. This held across raw, within-sample centered, and grand-mean centered specifications, as well as the preregistered specification that added radical attitudes and the interaction-focused CRF analyses. Because CRFs evaluate risk factors in direct competition and this null signal remained stable across different model specifications and samples, we can be confident that these group labels did not add meaningful explanatory value in this context.

## References

[B1] Ajzen I. (1991). The theory of planned behavior. *Organ. Behav. Hum. Decis. Process.* 50 179–211. 10.1016/0749-5978(91)90020-T

[B2] Ajzen I. Fishbein M. (1980). *Understanding Attitudes and Predicting Social Behavior.* Hoboken, NJ: Prentice-Hall.

[B3] Bandura A. (1999). Moral disengagement in the perpetration of inhumanities. *Pers. Soc. Psychol. Rev.* 3 193–209. 10.1207/s15327957pspr0303_3 15661671

[B4] Bélanger J. J. (2017). The rise and fall of violent extremism: The science behind community-based interventions. in *The Motivation-Cognition Interface: From the Lab to the Real World: A Festschrift in Honor of Arie W. Kruglanski*, eds Kopetz C. E. Fishbach A. (Milton Park: Routledge), 152–177. 10.4324/9781315171388-8

[B5] Bélanger J. J. (2021). The sociocognitive processes of ideological obsession: Review and policy implications. *Philos. Trans. R. Soc. B* 376:20200144. 10.1098/rstb.2020.0144 33612004 PMC7934954

[B6] Bélanger J. J. Adam-Troian J. Nisa C. F. Schumpe B. M. (2022). Ideological passion and violent activism: The moderating role of the significance quest. *Br. J. Psychol.* 113 917–937. 10.1111/bjop.12576 35678112

[B7] Bélanger J. J. Moyano M. Muhammad H. Richardson L. Lafrenière M.-A. K. McCaffery P. et al. (2019a). Radicalization leading to violence: A test of the 3N model. *Front. Psychiatry* 10:42. 10.3389/fpsyt.2019.00042 30853917 PMC6396731

[B8] Bélanger J. J. Nisa C. F. Schumpe B. M. Chamberland P. E. (2019b). Using implementation intentions to change passion: The role of environmental mastery and basic psychological needs. *Motiv. Sci.* 5 343–356. 10.1037/mot0000125

[B9] Bélanger J. J. Schumpe B. M. Nisa C. F. Moyano M. (2021). When countermessaging backfires: The role of obsessive passion in psychological reactance. *Motiv. Sci.* 7 83–95. 10.1037/mot0000206

[B10] Borenstein M. Higgins J. P. Hedges L. V. Rothstein H. R. (2017). Basics of meta-analysis: I2 is not an absolute measure of heterogeneity. *Res. Synthesis Methods* 8 5–18. 10.1002/jrsm.1230 28058794

[B11] Borum R. (2011). Radicalization into violent extremism I: A review of social science theories. *J. Strategic Security* 4 7–36. Available online at: https://digitalcommons.usf.edu/jss/vol4/iss4/2/

[B12] Della Porta D. (1995). *Social Movements, Political Violence, and the State: A Comparative Analysis of Italy and Germany.* Cambridge, MA: Cambridge University Press.

[B13] Doosje B. Loseman A. Van Den Bos K. (2013). Determinants of radicalization of islamic youth in the Netherlands: Personal uncertainty, perceived injustice, and perceived group threat. *J. Soc. Issues* 69 586–604. 10.1111/josi.12030

[B14] Doosje B. Moghaddam F. M. Kruglanski A. W. de Wolf A. Mann L. Feddes A. R. (2016). Terrorism, radicalization and de-radicalization. *Curr. Opin. Psychol.* 11 79–84. 10.1016/j.copsyc.2016.06.008

[B15] Doosje B. Van Den Bos K. Loseman A. Feddes A. R. Mann L. (2012). “My in-group is superior!”: Susceptibility for radical right-wing attitudes and behaviors in dutch youth. *Negotiation Conflict Manag. Res.* 5 253–268. 10.1111/j.1750-4716.2012.00099.x

[B16] Duval S. Tweedie R. (2000). Trim and fill: A simple funnel-plot-based method of testing and adjusting for publication bias in meta-analysis. *Biometrics* 56 455–463. 10.1111/j.0006-341X.2000.00455.x 10877304

[B17] Feigin B. Gómez Á Chinchilla J. Halperin E. (2026). When the target matters: A cross-cultural comparison of the generalizability and specificity of identity fusion effects. *Int. J. Intercult. Relat.* 113:102392. 10.1016/j.ijintrel.2026.102392

[B18] Geiger E. F. Brewster M. E. (2018). Development and evaluation of the individuals with learning disabilities and/or difficulties perceived discrimination scale. *Counseling Psychol.* 46 708–737. 10.1177/0011000018794919

[B19] Gollwitzer P. M. (1999). Implementation intentions: Strong effects of simple plans. *Am. Psychol.* 54 493–503. 10.1037/0003-066X.54.7.493

[B20] Gómez Á Bélanger J. J. Chinchilla J. Vázquez A. Schumpe B. M. Nisa C. F. et al. (2021). Admiration for Islamist groups encourages self-sacrifice through identity fusion. *Human. Soc. Sci. Commun.* 8 1–12. 10.1057/s41599-021-00734-9

[B21] Gómez Á Chinchilla J. Vázquez A. López-Rodríguez L. Paredes B. Martínez M. (2020). Recent advances, misconceptions, untested assumptions, and future research agenda for identity fusion theory. *Soc. Pers. Psychol. Compass* 14:e12531. 10.1111/spc3.12531

[B22] Gousse-Lessard A.-S. Vallerand R. J. Carbonneau N. Lafrenière M.-A. K. (2013). The role of passion in mainstream and radical behaviors: A look at environmental activism. *J. Environ. Psychol.* 35 18–29. 10.1016/j.jenvp.2013.03.003

[B23] Guimond S. Troian J. Ouoba N. M. Badea C. Nugier A. Bélanger J. J. (2025). Group relative deprivation and violent radicalism: Moving toward a comprehensive model. *J. Theoretical Soc. Psychol.* 2025:8582798. 10.1155/jts5/8582798

[B24] Gurr T. R. (1970). *Why Men Rebel.* Princeton, NJ: Princeton University Press.

[B25] Haslam N. Loughnan S. (2014). Dehumanization and infrahumanization. *Annu. Rev. Psychol.* 65 399–423. 10.1146/annurev-psych-010213-115045 23808915

[B26] Hilgard J. (2016). *PETPEESE* [Computer software]. GitHub. Available online at: https://github.com/Joe-Hilgard/PETPEESE

[B27] Horgan J. (2005). *The Psychology of Terrorism.* Milton Park: Routledge.

[B28] Imhoff R. Recker J. (2012). Differentiating Islamophobia: Introducing a new scale to measure Islamoprejudice and secular Islam critique. *Polit. Psychol.* 33 811–824. 10.1111/j.1467-9221.2012.00911.x

[B29] Institute for Economics and Peace. (2025). *Global Terrorism Index 2025: Measuring the Impact of Terrorism.* Available online at: https://www.visionofhumanity.org/wp-content/uploads/2025/03/Global-Terrorism-Index-2025.pdf (accessed January 15, 2026).

[B30] IntHout J. Ioannidis J. P. Rovers M. M. Goeman J. J. (2016). Plea for routinely presenting prediction intervals in meta-analysis. *BMJ Open* 6:e010247. 10.1136/bmjopen-2015-010247 27406637 PMC4947751

[B31] Jahnke S. Abad Borger K. Beelmann A. (2022). Predictors of political violence outcomes among young people: A systematic review and meta-analysis. *Polit. Psychol.* 43 111–129. 10.1111/pops.12743

[B32] Jugl I. Lösel F. Bender D. King S. (2021). Psychosocial prevention programs against radicalization and extremism: A meta-analysis of outcome evaluations. *Eur. J. Psychol. Appl. Legal Context* 13 37–46. 10.5093/ejpalc2021a6

[B33] Kassinove H. Sukhodolsky D. Tsytsarev S. V. Solovyova S. (1997). Self reported anger episodes in Russia and America. *J. Soc. Behav. Pers.* 12 301–324.

[B34] Kruglanski A. W. Bélanger J. J. Gunaratna R. (2019). *The Three Pillars of Radicalization: Needs, Narratives, and Networks.* Oxford: Oxford University Press.

[B35] Lösel F. King S. Bender D. Jugl I. (2018). Protective factors against extremism and violent radicalization: A systematic review of research. *Int. J. Dev. Sci.* 12 89–102. 10.3233/DEV-170241 31320326 PMC6703712

[B36] McCauley C. Moskalenko S. (2008). Mechanisms of political radicalization: Pathways toward terrorism. *Terrorism Polit. Violence* 20 415–433. 10.1080/09546550802073367

[B37] McCauley C. Moskalenko S. (2017). Understanding political radicalization: The two-pyramids model. *Am. Psychol.* 72 205–216. 10.1037/amp0000062 28383974

[B38] Moghaddam F. M. (2005). The staircase to terrorism: A psychological exploration. *Am. Psychol.* 60 161–169. 10.1037/0003-066X.60.2.161 15740448

[B39] Moskalenko S. McCauley C. (2009). Measuring political mobilization: The distinction between activism and radicalism. *Terrorism Polit. Violence* 21 239–260. 10.1080/09546550902765508

[B40] Obaidi M. Bergh R. Akrami N. Anjum G. (2019). Group-based relative deprivation explains endorsement of extremism among western-born muslims. *Psychol. Sci.* 30 596–605. 10.1177/0956797619834879 30875267

[B41] Peters J. L. Sutton A. J. Jones D. R. Abrams K. R. Rushton L. (2008). Contour-enhanced meta-analysis funnel plots help distinguish publication bias from other causes of asymmetry. *J. Clin. Epidemiol.* 61 991–996. 10.1016/j.jclinepi.2007.11.010 18538991

[B42] Pfundmair M. Leixner N. Pieper M. Frank R. Mehner R. Molinari S. et al. (2026). The pathway model of radicalization 2.0: Empirical insights into the religiously motivated, right-wing and left-wing radicalization process. *Terrorism Polit. Violence* 10.1080/09546553.2026.2646668 Advance online publication.

[B43] Rapoport D. C. (2013). “The four waves of modern terrorism,” in *An International History of Terrorism: Western and Non-Western Experiences*, eds Hanhimäki J. Blumenau B. (Milton Park: Routledge), 282–310.

[B44] Ribeaud D. Eisner M. (2010). Are moral disengagement, neutralisation techniques and self-serving cognitive distortions the same? Developing a unified scale of moral neutralisation of aggression. *Int. J. Conflict Violence* 4 298–315. 10.4119/ijcv-2833

[B45] Rip B. Vallerand R. J. Lafrenière M. A. K. (2012). Passion for a cause, passion for a creed: On ideological passion, identity threat, and extremism. *J. Pers.* 80 573–602. 10.1111/j.1467-6494.2011.00743.x 22091560

[B46] Rottweiler B. Gill P. (2022). Individual differences in personality moderate the effects of perceived group deprivation on violent extremism: Evidence from a United Kingdom nationally representative survey. *Front. Psychol.* 13:790770. 10.3389/fpsyg.2022.790770 35282250 PMC8908237

[B47] Rücker G. Schwarzer G. Carpenter J. R. Schumacher M. (2008). Undue reliance on I(2) in assessing heterogeneity may mislead. *BMC Med. Res. Methodol.* 8:79. 10.1186/1471-2288-8-79 19036172 PMC2648991

[B48] Schönbrodt F. D. Perugini M. (2013). At what sample size do correlations stabilize? *J. Res. Pers.* 47 609–612. 10.1016/j.jrp.2013.05.009

[B49] Snook D. W. Bouhelal A. Aidarov B. Zhabagin M. Abuov K. Bélanger J. J. (2025). By any means necessary: Utilitarian moral reasoning amplifies the connection between obsessive passion and ideological violence. *J. Community Appl. Soc. Psychol.* 35:e70159. 10.1002/casp.70159

[B50] Swann W. B. Jetten J. Gómez Á Whitehouse H. Bastian B. (2012). When group membership gets personal: A theory of identity fusion. *Psychol. Rev.* 119 441–456. 10.1037/a0028589 22642548

[B51] Vallerand R. J. (2015). *The Psychology of Passion: A Dualistic Model.* Oxford: Oxford University Press.

[B52] Vallerand R. J. Blanchard C. Mageau G. A. Koestner R. Ratelle C. Léonard M. et al. (2003). Les passions de l’ame: On obsessive and harmonious passion. *J. Pers. Soc. Psychol.* 85 756–767. 10.1037/0022-3514.85.4.756 14561128

[B53] Watson D. Clark L. A. Tellegen A. (1988). Development and validation of brief measures of positive and negative affect: The PANAS scales. *J. Pers. Soc. Psychol.* 54 1063–1070. 10.1037/0022-3514.54.6.1063 3397865

[B54] Webber D. Babush M. Morales C. Kucherenko N. Kruglanski A. W. (2018). The road to extremism: Field and experimental evidence that significance loss-induced need for closure fosters radicalization. *J. Pers. Soc. Psychol.* 114 270–285. 10.1037/pspi0000111 28872332

[B55] Weisburd D. Gill C. Wolfowicz M. Hasisi B. Litmanovitz Y. (2022). What is the best approach for preventing recruitment to terrorism? Findings from ABM experiments in social and situational prevention. *Criminol. Public Policy* 21 239–268. 10.1111/1745-9133.12579

[B56] Wilkes R. Wu C. (2019). Immigration, discrimination, and trust: A simply complex relationship. *Front. Sociol.* 4:32. 10.3389/fsoc.2019.00032 33869356 PMC8022697

[B57] Wolfowicz M. Litmanovitz Y. Weisburd D. Hasisi B. (2021). Cognitive and behavioral radicalization: A systematic review of the putative risk and protective factors. *Campbell Syst. Rev.* 17:e1174. 10.1002/cl2.1174 37133261 PMC10121227

[B58] Wong M. Y. Khiatani P. V. Chui W. H. (2019). Understanding youth activism and radicalism: Chinese values and socialization. *Soc. Sci. J.* 56 255–267. 10.1016/j.soscij.2018.08.006

