# Supplementary material for: An international tournament of risk factors for radical intentions: evidence from 40 countries
Source: Front Psychol. 2026 Sep 10;17:1855329. doi: 10.3389/fpsyg.2026.1855329 (PMC13600905; doi:10.3389/fpsyg.2026.1855329)
Supplement: Supplementary file 1 [file Supplementary_file_1.pdf]

Supplementary Information for

# An International Tournament of Risk Factors for Radical Intentions: Evidence From 40 Countries

## Supplemental Information A: Annotated Analyses

*Jocelyn J. Bélanger, Angel Gómez, Daniel W. Snook, Jais Adam-Troian, Michael Wolfowicz, Shadi Akariya, Jannis Kreienkamp, Justin König, Maximilian Agostini, Alexander P. Landry, N. Pontus Leander*

Corresponding Author: Jocelyn J. Bélanger

E-mail: jocelyn.belanger@gmail.com

Last updated: August 25, 2026

**Supplemental Information A: Annotated Analyses**

This supplementary information documents the methodology and results of all three studies. As part of our open supplemental materials, we share the full RMarkdown file which offers an annotated version of our all analysis steps. This file offers a transparent and reproducible analysis code, which includes full author commentary. We rendered the RMarkdown as an interactive HTML file, which we host as part of our open GitHub repository. We recommend the rendered version for almost all readers (full R code is also available via the rendered version).

https://thedataflowcompany.github.io/extremism-predictor-report/

For readers interested in the raw files, the raw RMarkdown file is available as part of the full GitHub repository (Bélanger et al., 2025).

## References

Bélanger, J. J., Gómez, A., Snook, D. W., Adam-Troian, J., Wolfowicz, M., Akariya, S., Kreienkamp, J., König, J., Agostini, M., Landry, A. P., & Leander, N. P. (2025). Extremism-predictor-project [github repository: Materials, computer code]. https://github.com/TheDataFlowCompany/extremism-predictor-project
